# Mononuclear Phagocyte-Derived Microparticulate Caspase-1 Induces Pulmonary Vascular Endothelial Cell Injury

**DOI:** 10.1371/journal.pone.0145607

**Published:** 2015-12-28

**Authors:** Srabani Mitra, Mark D. Wewers, Anasuya Sarkar

**Affiliations:** Davis Heart and Lung Research Institute, Division of Pulmonary, Allergy, Critical Care and Sleep Medicine, Department of Internal Medicine, The Ohio State University, Columbus, OH, United States of America; University of Technology Sydney, AUSTRALIA

## Abstract

Lung endothelial cell apoptosis and injury occurs throughout all stages of acute lung injury (ALI/ARDS) and impacts disease progression. Lung endothelial injury has traditionally been focused on the role of neutrophil trafficking to lung vascular integrin receptors induced by proinflammatory cytokine expression. Although much is known about the pathogenesis of cell injury and death in ALI/ARDS, gaps remain in our knowledge; as a result of which there is currently no effective pharmacologic therapy. Enzymes known as caspases are essential for completion of the apoptotic program and secretion of pro-inflammatory cytokines. We hypothesized that caspase-1 may serve as a key regulator of human pulmonary microvascular endothelial cell (HPMVEC) apoptosis in ALI/ARDS. Our recent experiments confirm that microparticles released from stimulated monocytic cells (THP1) induce lung endothelial cell apoptosis. Microparticles pretreated with the caspase-1 inhibitor, YVAD, or pan-caspase inhibitor, ZVAD, were unable to induce cell death of HPMVEC, suggesting the role of caspase-1 or its substrate in the induction of HPMVEC cell death. Neither un-induced microparticles (control) nor direct treatment with LPS induced apoptosis of HPMVEC. Further experiments showed that caspase-1 uptake into HPMVEC and the induction of HPMVEC apoptosis was facilitated by caspase-1 interactions with microparticulate vesicles. Altering vesicle integrity completely abrogated apoptosis of HPMVEC suggesting an encapsulation requirement for target cell uptake of active caspase-1. Taken together, we confirm that microparticle centered caspase-1 can play a regulator role in endothelial cell injury.

## Introduction

Lung vascular injury is a critical component of many insults that cause ALI/ARDS [1]. Although injury to the lung endothelium can occur by several mechanisms of which neutrophil-dependent injury is probably the most documented pathway, the detailed mechanisms leading to lung endothelial damage remain unclear. Recent published investigations have shown programmed cell death or apoptosis to be important factors in endothelial damage [2–6]. Some recent lines of evidence suggest the activation of Fas (CD95)/Fas ligand (FasL; CD178) system may play a pivotal role in lung vascular injury [7–9]. Despite increased awareness and investigation that provide insight into pathogenesis of cell injury and immune responses in ARDS, there are several gaps in our knowledge; as a result of which there is currently no effective pharmacologic therapy. As we begin to understand the mechanistic pathways responsible for vascular injury, the significance of inflammation in this process becomes irrefutable. Proinflammatory cytokines like TNFα, IL-1β and interferon IFNγ released by monocytes/macrophages have also been suggested to modulate cell apoptosis by regulating the expression of cell surface Fas and intracellular apoptosis-related proteins [10–13]. However, the actual mechanisms leading to injury remain incomplete and are likely to involve a combination of necrosis and apoptosis.

Microparticles/microvesicles (MPs/MVs) are released from cells on activation or during apoptosis as described in various pathological states, such as atherosclerosis, sepsis, acute coronary syndrome, diabetes or immune disorders [14–22]. Our previous studies have demonstrated monocyte-derived microparticles to be involved in apoptosis and cell loss in sepsis [23, 24]**.** The findings suggest that microparticulate caspase-1 released during sepsis is important in the host response to sepsis, at least in part, via its ability to induce apoptosis. Microparticles have also been shown to have pathological consequences on organ injury [16–18]. However, little is known about the effects of these microparticles on endothelium integrity and lung vascular injury.

IL-1β and IL-18, proinflammatory cytokines regulated by caspase-1 have been implicated in various diseases conditions septic shock, inflammatory bowel disease, diabetes mellitus, rheumatoid arthritis and myocardial disease [25–32]. Our recent work establishes the involvement of caspase-1 in NF-κB and apoptosis regulation [33, 34]. The discovery of novel connection of caspase-1 to the inflammatory and NF-κB signaling cascade, thereby regulating apoptosis provide further evidence that this is an area of critical significance. Furthermore, we and others have shown that the blockade of this apoptosis with the broad caspase inhibitor ZVAD-fmk improves mortality in septic mice [34, 35], suggesting the role of caspases in induction of apoptosis. Importantly, we have recently documented that caspase-1 can be released from mononuclear phagocytes in a microparticulate encapsulated form.

We therefore, hypothesize that MPs serve to package and deliver active caspase-1 to endothelial cells inducing injury and apoptosis characteristic of ALI/ARDS. To test this hypothesis, we chose to analyze the role of monocyte/THP1 derived microparticles in lung injury from a caspase perspective.

## Materials and Methods

### Reagents

Lipopolysaccharide (LPS) from *Escherichia coli* strain 0111:B4 was obtained from Enzo Life Sciences (Plymouth,PA). RPMI 1640 was purchased from Mediatech Inc. (Manassas,VA) and phosphate buffered saline (PBS) from Life Technologies (Grand Island, NY)., and fetal bovine serum (FBS) from Atlas Biologicals (Fort Collins, CO). The pan-caspase inhibitor, z-Val-Ala-Asp (O-Methyl) fluoromethyl ketone (zVADfmk) and IL-1β Converting Enzyme (ICE) Inhibitor II (Ac-YVAD-CMK) were purchased from EMD Biosciences (San Diego,CA). The phospholipid membrane dye, lipophilic carbocyanine DilC16(3) (D384,1.25uM) was purchased from Life Technologies. A limulus amebocyte lysate assay kit (LAL) was purchased from Lonza (Walkersville, MD). All other reagents were obtained from Sigma-Aldrich (St. Louis, MO) unless otherwise specified.

### THP1, primary monocytes and endothelial cell culture conditions

Human THP1 monocytic cells, obtained from ATCC, were cultured in RPMI 1640 supplemented with 10% fetal bovine serum (FBS) at 37°C in a humidified CO_2_ incubator. Primary monocytes were isolated from Red Cross buffy coats as described in [23]. Primary human pulmonary microvascular endothelial cells (HPMVECs) were obtained from Lonza (Walkersville, MD). They were maintained in EGM2 MV Bulletkit System (Lonza). HPMVECs were co-cultured with either microvesicles or conditioned medium as per further experiments. Co-culture experiments were performed in RPMI medium with 10% FBS throughout the experiments.

### Microparticle isolation

Microparticle isolation was performed following our published procedure [23]. Briefly, supernatants from THP1 stimulated or not with LPS (1μg/ml) for 2h were subjected to serial centrifugation at 2000 g for 5 min, 16,000 g for 5 min to remove cell debris and aggregates and finally ultracentrifuged at 100,000 g for 1h [23, 36–43] to isolate microparticles. Microparticles were then resuspended in PBS and subjected to characterization using flow cytometry as described in [23]. Microparticles were also stained with phospholipid dye, DiLC16, for visualization under fluorescence microscope for uptake assays [44, 45]. Briefly, THP1 cells were incubated with the dye for 15min, washed with PBS and then stimulated with LPS for 2h as described previously. MPs were isolated and observed under fluorescence microscopy for confirmation of staining. Functional studies were then performed with the pelleted MPs by subjecting them to immunoblotting, caspase-1 enzymatic assay or coculture assays with endothelial cells (HPMVECs).

### Cell death assays

Endothelial cells cultured at a confluency of 60–70% were subjected to microparticles released from THP1 in the presence or absence of inhibitors. First HPMVECs were subjected to cell death analysis using MTS assay. The MTS assay is based on the conversion of a tetrazolium salt into a colored aqueous soluble formazan product by mitochondrial activity of viable cells at 37°C. The amount of colored product formed is directly proportional to the number of living cells in culture measured at 490nm. The detection reagent used in his study was purchased from Promega (Madison, WI). In this study the endothelial cells seeded in a 12 well tissue culture plate were treated with either conditioned media or microparticles in a CO_2_ incubator at 37°C overnight. Medium was removed the following day, cells washed three times with RPMI medium containing 10% FBS. The MTS reagent was added to the cells in a ratio of 1:5 (Reagent mixture: culture medium) and incubated for 1h at 37°C in a CO_2_ incubator. The absorbance was measured in a plate reader 490nm (Perkin Elmer 2030 Multilabel Reader, Shelton CT). Secondly, cell death was also analyzed by light microscopy based on standard characteristic apoptotic features like shrunken, compacted nuclei (pyknosis) and/or nuclear fragmentation (karyorrhexis). Random ten fields from the slides were examined at 100x magnification. Finally, endothelial cell death/apoptosis was also confirmed using annexin V/PI detection kits purchased from BD Biosciences, (San Diego, CA) following the manufacturer’s protocol (as a marker for apoptosis).

### Caspase-1 activity assay and immunoblot

Total caspase-1 and caspase-1 enzymatic function was measured as previously described [23]. Briefly, supernatants from THP1 monocytic cells, as well as microparticles were subjected to caspase-1 ELISA or enzymatic assay. For enzymatic assays, 50μl sample was mixed with 50μl of assay buffer {(50mM HEPES (pH 7.4), 100 mM NaCl, 0.1% 3-[(3-cholamidopropyl)dimethylammonio]-1-propanesulfonate, 20% glycerol, 10mM DTT and 0.1mM EDTA) and 5μl of 1mM Ac-WEHD-AMC} and subjected to kinetic fluorometric assay using a Cytofluor 4000 fluorometer (Perspective, Framingham, MA) at 360nm excitation and 460nm emission [46,47]. Presence of caspase-1 in microparticles and other fractions was also confirmed by immunoblot using caspase-1 rabbit polyclonal antibody generated by our laboratory as the primary antibody and donkey anti rabbit from GE Healthcare (UK) as the secondary antibody. Protein amounts were all normalized during loading for comparison purposes.

### Limulus Amebocyte Lysate Assay (LAL Assay)

A limulus amebocyte lysate assay kit for the evaluation of endotoxin in the samples was purchased from Lonza (Walkersville, MD). Endotoxin measurement in the samples was performed as per the manufacturer’s recommendations.

### Statistical analysis

Data are represented as the mean ± standard error of the mean (SEM) from at least three independent experiments. All other comparisons were performed with ANOVA, followed by post hoc comparison analysis using Tukey-Kramer HSD. p<0.05 was considered to represent statistical significance.

## Results

### THP1 released microparticles induced endothelial cell death

**Fig 1:**
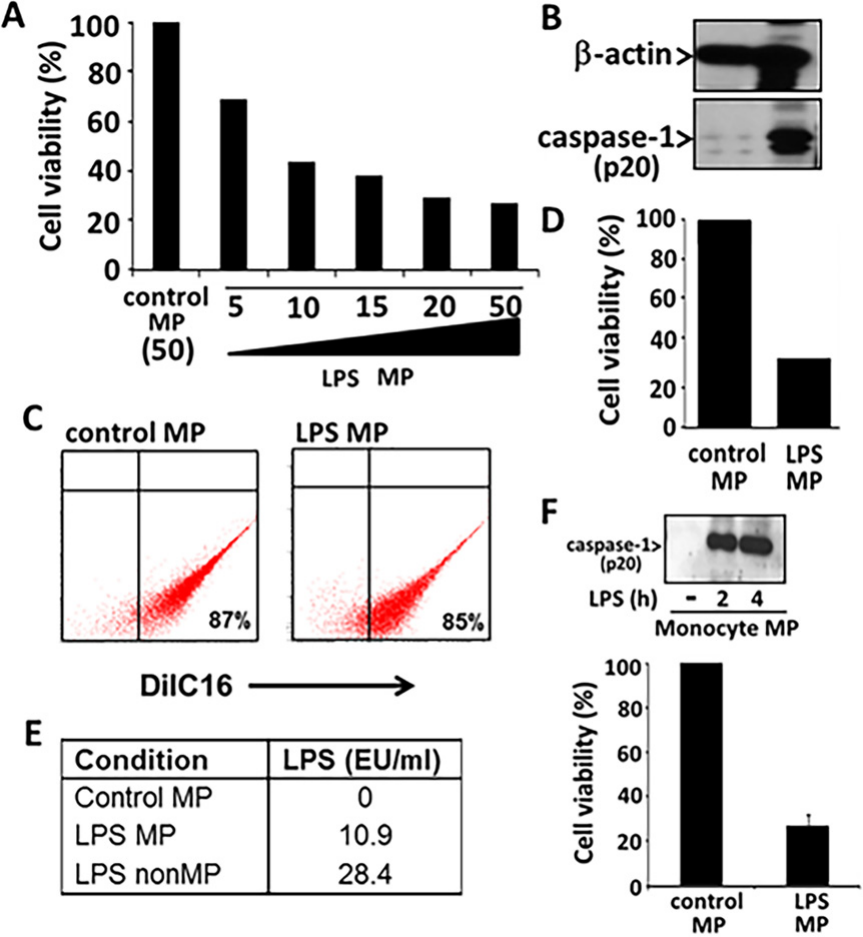
Microparticles released from monocytic cell THP1 induce human pulmonary microvascular endothelial cell (HPMVEC) death. THP1 cells were cultured at 5,10,15,20 and 50 million/ml and stimulated with LPS (1μg/ml) for 2h or left untreated. (A) Microparticles (MP) were isolated from each condition and subjected to HPMVEC and analyzed for cell death using MTS assay. (B) MPs were also subjected to careful normalization by total protein prior to subjecting to HPMVEC. (B) Immunoblot analysis of both control MP from 50X10^6^ THP1/ml and LPS MP from 10X10^6^ THP1/ml, normalized based on total protein content was analyzed for β-actin (loading control) and presence of active caspase-1 (p20). (C) Normalized MP fractions (stained with DilC16) were also analyzed by flow cytometry. (D) These MP fractions were then subjected to HMPVEC and analyzed for cell death by MTS assay. (E) LPS amounts were measured from MPs isolated from unstimulated THP1 (50X10^6^ THP1/ml) and LPS stimulated THP1 (10X10^6^ THP1/ml) and nonMP fractions using LAL assay. (F) MPs were also isolated from primary monocytes from Red Cross buffy coats. Monocytes were isolated from buffy coats and stimulated with LPS similar to THP1 cells for 2 and 4 h. Isolated MPs were analyzed for presence of active caspase-1 (p20) and induction of HPMVEC cell death (MPs form 2h stimulation used for MTS assay). Quantitative analysis for n = 2 experiments.

We analyzed LPS stimulated monocytic cell line (THP1) released microparticles for their ability to regulate HPMVEC death. Primary human pulmonary microvascular endothelial cells (HPMVEC) were cocultured with THP1 supernatants and its fractions in the presence or absence of LPS (1μg/ml) for 24 h. THP1 cells were stimulated with LPS (1μg/ml) for 2h and then supernatant was separated from the cell fraction as described previously. The supernatant fraction was then further separated into microparticles (MP) and non-microparticulate fractions (nonMP) by serial centrifugation and ultracentrifugation. Micropartciles (MPs) released by THP1 cells were quantified by staining with DilC16 for normalization purposes. Microparticles were normalized by calibrating the dosage of MPs added to the endothelial cells. MPs generated from 5,10,15,20 and 50 X10^6^ LPS stimulated THPs were compared to control MPs from 50 million unstimulated THPs for induction of apoptosis. Based on the MTS assay and protein amounts, MPs from 10 million stimulated THP1 were comparable in total protein amount to control MPs from 50 million unstimulated THPs and induced about 58% cell death (Fig 1A). Total protein measurements in these samples were 677μg/ml for control MP and 680 μg/ml for LPS MP. MP fractions were then stained with membrane dye DilC16 and then subjected to flow cytometry analysis for quantification. Control and LPS MP were 87% and 85% positive MPs respectively (Fig 1C). We further subjected these fraction s to immunoblot analysis for protein quantification. As shown in Fig 1B, both control and LPS MPs normalized by total protein had equal amounts of β-actin. However, only the LPS MP fraction had active caspase-1 (p20). Having confirmed that the amounts of MPs from both control and LPS samples were comparable, we then subjected them to HPMVEC and analyzed for cell death. As shown in Fig 1D, only LPS MPs induced cell death of HPMVECs (71%) in comparison to control MPs. These normalized concentrations of MPs were used for future experiments.

**Fig 2:**
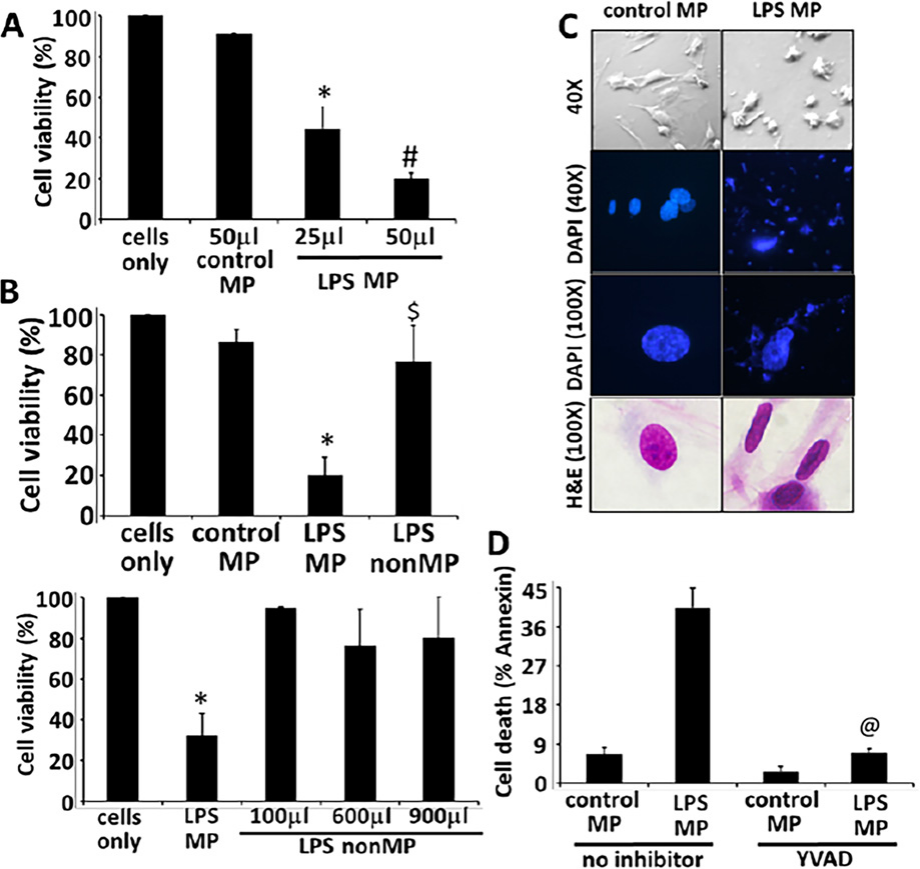
Human pulmonary microvascular endothelial cell apoptosis is mediated by THP-1 stimulated microparticles and inhibited by caspase-1 inhibitor. THP1 cells were stimulated with LPS (1μg/ml) for 2h and supernatants were separated into microparticle (MP) and non- microparticle (nonMP) fractions by serial centrifugations. HPMVECs were co-cultured with these MP and nonMP fractions and analyzed for cell viability and apoptosis. Cell viability was measured by MTS assay (A and B) and morphology using DAPI staining (C). HPMVECs were then treated with microparticles isolated from unstimulated or LPS induced THP1 in the presence or absence of the caspase-1 inhibitor, YVADcmk (D). Apoptosis was analyzed using Annexin V/PI assay by flow cytometry. Quantitative analysis for n = 3 experiments. * #Comparison of LPS MP to control MP/cells only, $ Comparison of LPS MP to LPS nonMP, @ Comparison of LPS MPs, with and without YVAD.

Microparticles from THP1 stimulated with LPS (LPS MP) were then cultured with HPMVEC which induced significant HPVEC cell death (60%), as compared to endothelial cells co-cultured with control unstimulated microparticles (control MP) (p<0.0001) (Fig 2A). In contrast, non-microparticulate fractions (non-MP) were unable to induce cell death (Fig 2B). THP1 non-MPs were normalized by volume. THP1 cells were stimulated in 1ml volume and the entire volume of nonMP fraction after untracentrifuge (1ml) was used to perform the experiments. Cell death was also confirmed by light microscopy at 40X and 100x magnification and apoptotic features were recorded using DAPI staining (Fig 2C). Finally, cell death was confirmed to be apoptotic by Annexin V/PI analysis. HPMVEC co-cultured with LPS MP showed 40% of cells positive for Annexin V staining, as compared to 5% for control MP fractions (p<0.001) (Fig 2D). Microparticles from THP1 cells pretreated with the caspase-1 inhibitor YVAD before LPS challenge were unable to induce cell death of HPMVEC, suggesting a role for caspase-1 or its substrate in the induction of HPMVEC cell death. A similar apoptotic role for MPs was also observed from primary monocytes. MPs from LPS (1μg/ml) stimulated primary monocytes induced approximately 70% cell death of HPMVEC by MTS analysis (Fig 1F).

### Microparticulate caspase-1 induces vascular endothelial cell apoptosis

**Fig 3:**
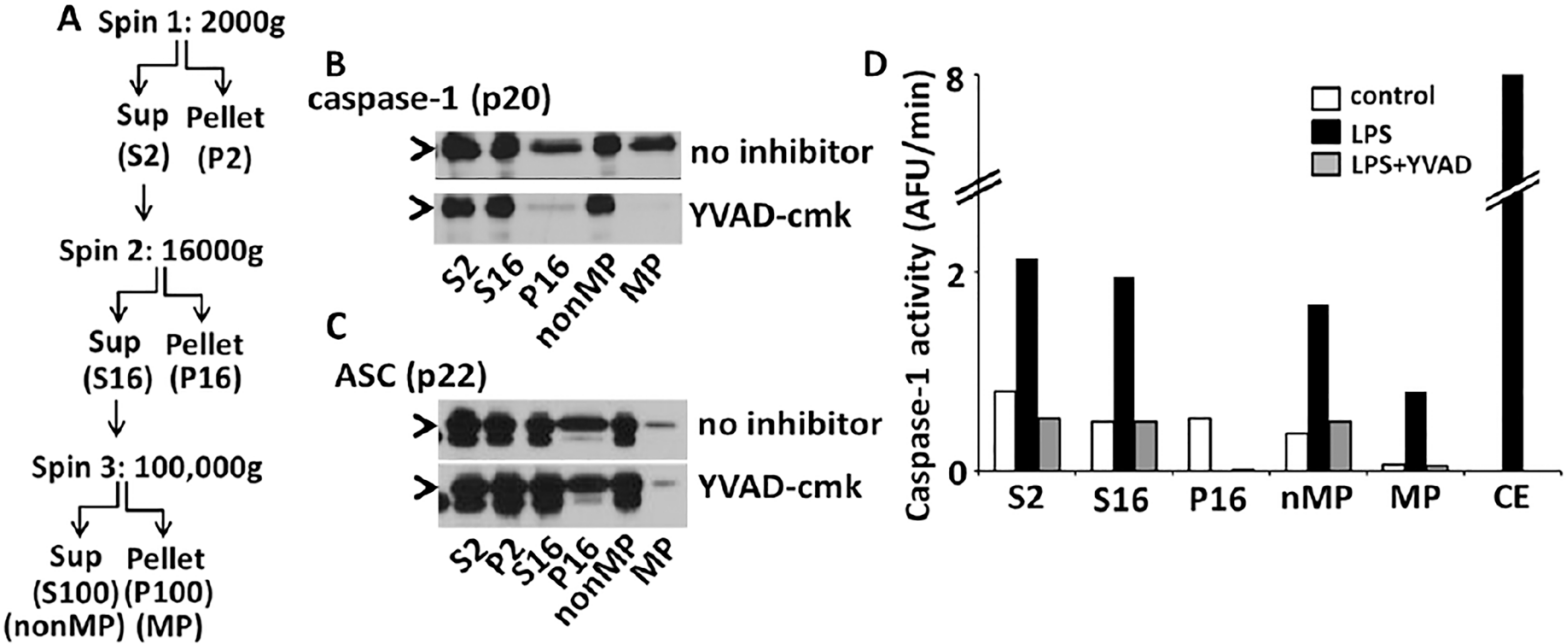
Active caspase-1 and ASC release in microparticles from stimulated THP-1. THP1 cells were stimulated with LPS (1μg/ml) for 2h in the presence or absence of YVAD-cmk. Microparticles were isolated from LPS stimulated THP-1 supernatant by stepwise ultracentrifugation (A). The cell fraction was separated by centrifugation at 2000 g for 5 min. Supernatants were then further fractionated into a 16,000g fraction followed by microparticle (MP) fraction and non- microparticle (nonMP) fraction by a final ultracentrifugation at 100,000 g for 1h. Microparticles (MP) and non-microparticle fractions (nMP) were analyzed for presence of the p20 form of caspase-1 (B) and the inflammasome adaptor protein, ASC (C) by immunoblot. Substrate cleavage capacity of caspase-1 from each of the supernatant fraction was also measured using WEHD-enzymatic assay (D). Analysis for n = 2 experiments.

To determine if the microparticle mediated endothelial cell death was in fact caused by active caspase-1, HPMVEC were co-cultured with MP fractions of LPS stimulated THP1 cells pretreated or not with the caspase-1 specific inhibitor, YVAD-cmk for 30min prior to stimulation. Microparticles from THP1 pretreated with YVAD-cmk before LPS challenge were unable to induce HPMVEC cell death, suggesting the role of caspase-1 or its substrate in the induction of cell death (Fig 2D and Table 1). The non-particulate fractions (nonMP) did not kill the endothelial cells. Corroborating the YVAD data, microparticles from THP1 cells pretreated with the pancaspase inhibitor, ZVAD-fmk completely abrogated the MP-induced cell death (78±5% survival as compared to 37±9% from untreated LPS-MVs) (Table 1). This protective effect of caspase-1 inhibition on endothelial cell death correlated with the absence of the active caspase-1 form (p20) in the MV fractions pretreated with YVAD (Fig 3). Processed p20 caspase-1 was released from THP1 cells stimulated with LPS within 2h. More importantly, p20 caspase-1 was detected in the supernatant microparticle fraction (Fig 3B **)**. The caspase-1 released in the MPs was confirmed to be active by WEHDafc assay (Fig 3D). Pretreating THP1 cells with the caspase-1 inhibitor, YVADcmk, completely abrogated the LPS induced release of active caspase-1 (p20) in the MPs (Fig 3B). The inhibitor also completely prevented p20 caspase-1 (active form) detection in the MP fractions as well as cell extract, supernatant and nonMP fractions (Fig 3D). Of note, MPs also contained the inflammasome protein ASC, critical for caspase-1 activation. ASC, similar to caspase-1, was identified both in the supernatants and microparticles from LPS stimulated THP1s (Fig 3C). Caspase-1 inhibition did not prevent ASC presence in the microparticles.

**Table 1:** Effect of caspase-1 inhibition on microparticle-induced HPMVEC apoptosis.

Condition	Additional Treatment	Cell survival (%)
No MP	None	100
Control MP	None	85±5 *
LPS-induced MP	None	37±9 * #
LPS-induced MP	YVAD to THP1	87±9 #
LPS-induced MP	YVAD to MP	71±9 #
LPS-induced MP	YVAD to HPMVEC	89±27 #
LPS-induced MP	ZVAD to THP1	77±7 #
LPS-induced MP	ZVAD to MP	78±5 #
LPS-induced MP	ZVAD to HPMVEC	78±14 #
No MP	YVAD	85±26
No MP	ZVAD	90±24
No MP	LPS	90±17

THP1 cells were stimulated with LPS (1μg/ml) for 2h and microparticles (MP) were isolated. MPs were generated from cells in the presence or absence of specific caspase-1 inhibitor, YVAD-cmk or pan-caspase inhibitor, ZVAD-fmk. Inhibitors were added either A) to the cells prior to LPS stimulation (pre-generation); B) to the LPS stimulated MPs directly after isolation from the supernatant (post-generation) or C) to the endothelial cells (HPMVEC) directly prior to exposure to LPS MPs. HPMVECs from all conditions were co-cultured overnight and analyzed for cell viability by MTS assay. LPS and inhibitors were also added separately to the HPMVECs directly and analyzed for cell viability.

* p<0.05 when compared between unstimulated MP and LPS MP

# p<0.05 when compared between LPS MP with and without inhibitors. Data represent means ± SEM, n = 3.

To confirm that it is the exogenous caspase-1 and not the endogenous HPMVEC caspase-1 inducing HPMVEC death, endothelial cells were pretreated with YVAD-cmk for 30min and then washed to remove extracellular YVAD prior to co-culture with LPS MV. Alternatively, microparticles isolated from THP1 cells after LPS stimulation (i.e., containing activated caspase-1) were also treated with YVAD for 30min (post generation) and then washed to remove the non-particle associated inhibitor before subjecting them to endothelial cells. Both pre-treatment of target endothelial cells with YVAD or treating LPS MP with caspase-1 inhibitor, YVAD or pan-caspase inhibitor, ZVAD post-generation, abrogated endothelial cell death by approximately 50% compared to untreated LPS MP (Table 1).

To further confirm that the effect on HPMVEC represents microparticulate caspase-1 and not simply an endotoxin effect, we have used several approaches while preparing the microparticles. Firstly, MPs were subjected to several spins, including ultracentrifugation followed by washing with media before subjecting them to endothelial cells. Secondly, we have added LPS (1μg/ml) directly to the endothelial cells as a control. LPS was also added to control MPs prior to being subjected to HPMVEC. No HPMVEC killing was observed either upon direct challenge with LPS or with LPS added to control MPs. Finally, LPS amounts were measured from control MP, LPS MP and LPS nonMP fraction using Limulus Amebocyte Lysate Assay (LAL) assay system from Lonza (Walkersville, MD) per the manufacturer’s recommendations. We have previously shown in Fig 2B that nonMPs are unable to induce cell death in contrast to MPs. By LAL assay, we observed that LPS MPs had 10.9 EU/ml vs higher amounts of LPS in nonMP fractions (28.4EU/ml) upon normalization (Fig 1E). Together, these studies indicate that the effects seen by MPs were not due to contaminating endotoxin.

### Encapsulation of exogenous caspase-1 is essential for its apoptotic function

**Fig 4:**
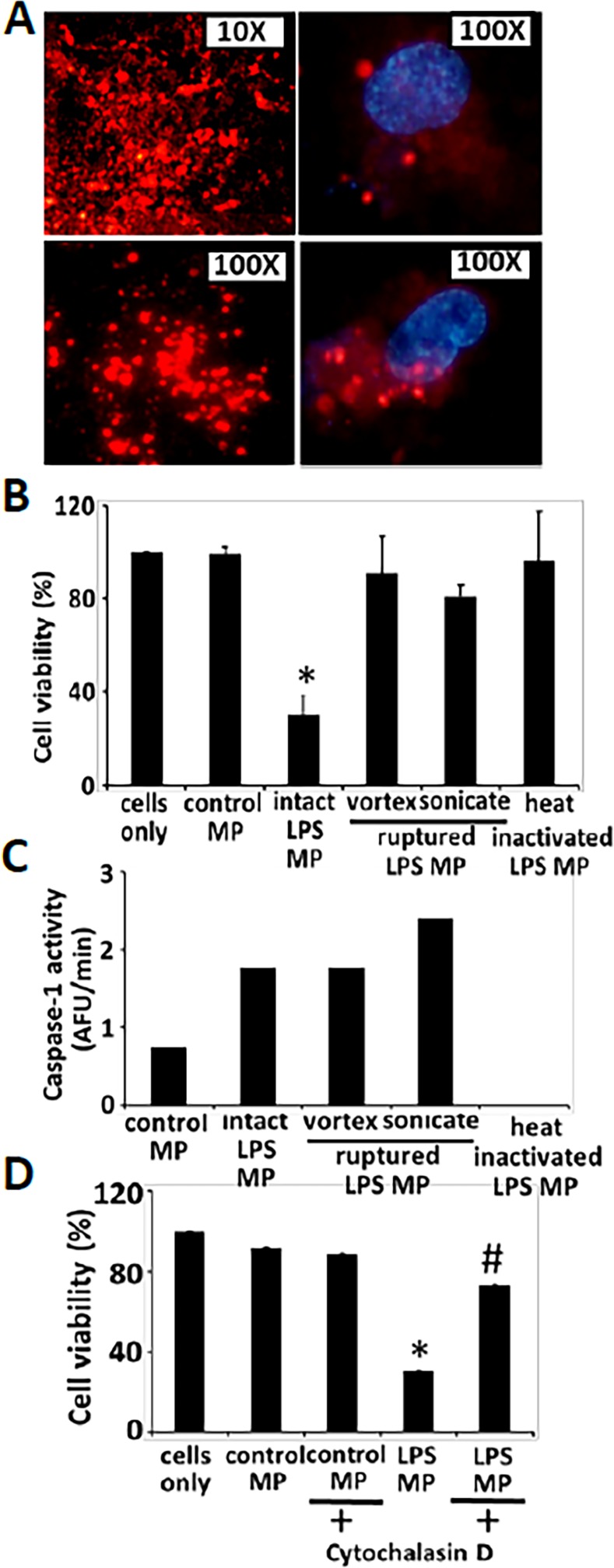
Encapsulation is necessary for exogenous caspase-1 mediated apoptosis. THP1 cells were first labeled with phospholipid dye, DiLC16 for 30 min, washed, stimulated with LPS (1μg/ml) for 2h and microparticles were isolated. A) Labeled microparticles were observed under fluorescence microscope; left panel (top and bottom). Uptake of labeled microparticles by DAPI stained HPMVEC was observed by fluorescence microscopy (right panel: control MP (top) and LPS MP (bottom)). B) Microparticles were then either kept intact or disrupted by vortexing or by sonication, or subjected to heat inactivation. Microparticles were then applied to HPMVECs and endothelial cell viability was analyzed by MTS assay (n = 3). * intact LPS MP vs control MP or intact LPS MP vs ruptured/ heat inactivated LPS MPs. C) Caspase-1 activity of each fraction was measured using WEHD enzymatic assay from two experiments. Mild homogenization of microparticles or sonication did not affect caspase-1 activity in contrast to heat inactivation. D) HPMVEC were pretreated with cytochalasin D (5μg/ml) for 30 min, washed two times to remove trace of reagent and then control or LPS MP was subjected to cells as previously described. Uptake of microparticles and effect on apoptosis of HPMVEC was analyzed by MTS assay (n = 3 experiments) * intact LPS MP vs control MP; # LPS MP with cytochalasin D vs LPS MP.

Finally, we examined the role of encapsulation in the apoptotic function of exogenous caspase-1. To test this hypothesis, uptake of DiLC16 labeled microparticles derived from LPS stimulated THP1 (as described in materials and method) were first characterized by fluorescent microscopy. Briefly, MPs were isolated from DiLC16 labeled THP1s, washed twice (by ultracentrifugation) to remove any excess DiLC16 and then added to healthy HPMVEC for 12h on coverslips. Cells attached to the coverslips were then washed twice gently and mounted on slides to analyze for uptake by DAPI stained HPMVEC under fluorescent microscopy (Fig 4A). DiLC16 labeled microparticles were then either kept intact or subjected to disruption by vortexing or sonication (5 pulses X 3 on ice) or heat-inactivation (incubated at 100°C for 10 min). Endothelial cells were then co-cultured with these MPs and analyzed for cell viability by MTS assay. Intact microparticles from LPS stimulated THP1 (LPS MP) induced significant cell death (23% viability as compared to cells exposed to control MP) (Fig 4B). In contrast, cell death was abrogated in cells exposed to ruptured or heat-inactivated LPS MP (90% survival). Intact, homogenized and heat-inactivated microparticles were then analyzed for caspase-1 activity. Both intact and ruptured microparticle fractions contained active caspase-1, whereas the caspase-1 activity was completely lost by heat-inactivation of microparticles (Fig 4C). Caspase-1 activity in LPS MP was significantly higher than control MP (0.5 AFU/min for control MP vs 1.8 AFU/min for LPS MP). That microparticle disruption maintained caspase-1 enzymatic function but lost cell death activity, suggest that microparticle encapsulation of active caspase-1 is critical for the cell death induction. Furthermore, to test whether uptake of encapsulated MPs are essential for its apoptotic function, endothelial cells were pretreated with cytochalasin D for 30 min, washed twice to remove cytochalasin D and then subjected to intact LPS MP. Cytochalasin D completely abrogated the uptake of intact microparticles (LPS MP) which inhibited cell death (30% cell viability in LPS MP vs 73% cell survival in cytochalasin D treated cells, p<0.024) (Fig 4D), suggesting that uptake of microparticle encapsulated exogenous caspase-1 is essential for its apoptotic function.

## Discussion

Despite remarkable advances in ARDS/ALI research highlighting the many facets of innate immunity, our understanding of the complexity and critical importance of innate responses in the context of infectious challenges and acute lung injury is rudimentary. Endothelial cell (HPMVEC) damage and apoptosis occurs in almost all initial stages of ALI/ARDS and is triggered by a combination of factors and conditions [2–13]. Endothelial cell apoptosis is an important factor throughout all stages of lung injury. Because circulating mononuclear phagocytes, monocytes are key circulating inflammatory cells that are reservoirs of caspase-1, which has a role in both inflammatory cytokine processing and apoptosis, we hypothesized that monocyte/macrophages release caspase-1 in target specific microparticles that regulate the severity of endothelial injury and dysfunction.

The role of caspase-1 in regulation of apoptosis although recently appreciated is still controversial, in spite of the fact that caspase-related protease family members been shown to play an important role in apoptosis [48–51]. It has been well documented that caspase-1 knockouts are completely healthy in contrast to caspase-3 deficient animals which have major birth defects implying caspase-3’s role in apoptosis in developmental stage [51,52]. Previous published work from our own laboratory demonstrates that spontaneous monocyte apoptosis is not dependent upon caspase-1 but upon caspase-3 activity [53]. Caspase-1 has been implicated in regulation of apoptosis in rat fibroblast cell line [46], neuronal cell apoptosis [54], as well as in Salmonella infected dendritic cells and monocyte derived macrophages [55–57]. In our own published work [23,24,34], we have demonstrated the novel role of caspase-1 in lymphocyte apoptosis and sepsis survival and smooth muscle cells apoptosis in atherosclerosis.

Our *in vitro* model of endothelial cell (HPMVEC) monocyte interactions was developed to first study the potential role of microparticulate caspase-1 as a means to vascular injury. This novel pathway has not been previously studied and hence offers a unique opportunity to improve our understanding of the pathophysiology of ARDS. We describe for the first time the novel role of monocyte-derived, microvesicular caspase-1 in the induction of apoptosis of HPMVECs. Microparticles (MP) are known to carry different factors and proteins upon being shed by cells upon activation or apoptosis [14,15]. For example, monocyte/macrophage derived microparticles have been shown to transport phosphatidyl serine and tissue factor (TF) [36–43]. In the present work and previous studies by our laboratory, we provide clear evidence of packaging of exogenous caspase-1 into microparticles [23, 24]. We also demonstrate that endothelial death was induced by active caspase-1 released by activated THP1s in microparticles, whereas, HPMVEC cultured with LPS directly induced no significant cell death. Apoptosis induced by microparticulate caspase-1 was completely abrogated by the specific caspase-1 inhibitor, YVAD-cmk. That apoptosis was also inhibited upon rupturing the microparticles by mechanical disruption, heat inactivation or by cytochalasin D, suggests that encapsulation of caspase-1 is essential for uptake and apoptotic function. We have previously demonstrated that caspase-1 release in microparticles parallels the release of its substrate IL-1β and IL-18 [23]. IL-1β release has already been hypothesized to occur in the form of small packages termed microvesicles or vesicular bodies [40–42, 47]. The present study further confirms our observation that active caspase-1 is released in microparticles by activated THP1s and that this encapsulation is critical for uptake and induction of endothelial apoptosis. Furthermore, we have also identified the presence of ASC, an inflammasome adaptor protein critical for caspase-1 activation to be released in microparticles along with caspase-1.

In summary, this present works provides evidence that caspase-1 plays a crucial role in regulating apoptosis of pulmonary vascular endothelial cells, which may have particular relevance for sepsis-related vascular injury. This novel apoptotic function of caspase-1 is dependent upon encapsulation of exogenous caspase-1 into circulating microparticles that allow uptake of the active enzyme by vascular endothelial cells.
